# A multi-centre, randomized, double-blind, placebo-controlled clinical trial of the efficacy and safety of chloroquine phosphate, hydroxychloroquine sulphate and lopinavir/ritonavir for the treatment of COVID-19 in Lagos State: study protocol for a randomized controlled trial

**DOI:** 10.1186/s13063-021-05675-x

**Published:** 2021-12-04

**Authors:** A. Abayomi, A. Osibogun, O. Ezechi, K. Wright, B. Ola, O. Ojo, Y. Kuyinu, E. Zamba, H. Abdur-Razzaq, O. A. Erinoso, S. E. Anya

**Affiliations:** 1 Lagos State Ministry of Health, Alausa, Ikeja, Lagos State Nigeria; 2 Lagos State Primary Health Care Board, Lagos, Nigeria; 3 Nigerian Institute of Medical Research, Lagos, Nigeria; 4 Lagos State University College of Medicine, Lagos, Nigeria; 5 Lagos State University Teaching Hospital, Lagos, Nigeria; 6 Lagos State Health Management Agency, Lagos, Nigeria

**Keywords:** COVID-19, SARS-CoV-2, 2019-nCoV, Chloroquine, Hydroxychloroquine, Lopinavir/ritonavir, Lagos, Nigeria

## Abstract

**Background:**

The coronavirus disease 2019 (COVID-19) is caused by the severe acute respiratory syndrome coronavirus 2 (SARS-CoV-2) that was first identified in Wuhan, Hubei, China, in December 2019. It was recognized as a pandemic by the World Health Organization on 11 March 2020. Outbreak forecasting and mathematical modelling suggest that these numbers will continue to rise. Early identification of effective remedies that can shorten the duration and severity of illness is critical for Lagos State, which is the epi-centre of the disease in Nigeria.

**Methods:**

This is a multi-centre, randomized, double-blind placebo-controlled superiority trial. The study investigates the efficacy of chloroquine phosphate, hydroxychloroquine sulphate and lopinavir/ritonavir added on to standard of care compared to standard of care only in patients with COVID-19 disease. The primary outcome is the clinical status of patients measured using a 7-point ordinal scale at day 15. Research participants and clinicians will be blinded to the allocated intervention. Outcome measures will be directly assessed by clinicians. Statistical analysis will be done by a team blinded to the identity and allocation of research participants. Data analysis will follow intention-to-treat methods, using R software.

**Discussion:**

The current study is of strategic importance for Lagos State in potentially curbing the health, social and economic burden of COVID-19 disease. Should the current study demonstrate that either of the three intervention drugs is more efficacious than standard therapy alone, the State Ministry of Health will develop an evidence-based guideline for the management of COVID-19 in Lagos State. The findings will also be shared nationally and with other states which may lead to a standardized national guideline for the treatment of COVID-19 in Nigeria.

**Trial registration:**

Pan African Clinical Trials Register PACTR202004801273802. Registered prospectively on April 2, 2020

**Supplementary Information:**

The online version contains supplementary material available at 10.1186/s13063-021-05675-x.

## Administrative information

Note: the numbers in curly brackets in this protocol refer to SPIRIT checklist item numbers. The order of the items has been modified to group similar items (see http://www.equator-network.org/reporting-guidelines/spirit-2013-statement-defining-standard-protocol-items-for-clinical-trials/) (Table 1).

**Table 1:** Administrative information for Lagos COVID-19 Treatment Trial (LACTT)

Title {1}	A Multi-Center, Randomized, Double-Blind, Placebo-Controlled Clinical Trial of the Efficacy and Safety of Chloroquine Phosphate, Hydroxychloroquine Sulphate and Lopinavir/Ritonavir for the Treatment of COVID-19 in Lagos State, Nigeria
Trial registration {2a and 2b}.	The trial was registered with the Pan African Clinical Trials Register on April 2nd, 2020 with registration number PACTR202004801273802. The URL of the registry record is https://pactr.samrc.ac.za/TrialDisplay.aspx?TrialID=10928.
Protocol version {3}	20^th^ July 2020, version number: 3.0.
Funding {4}	Lagos State Government
Author details {5a}	^1^Lagos State Ministry of Health, Lagos, Nigeria ^2^Lagos State Primary Health Care Board, Lagos, Nigeria ^3^Nigerian Institute of Medical Research, Lagos, Nigeria ^4^Lagos State University College of Medicine, Lagos, Nigeria ^5^Lagos State University Teaching Hospital, Lagos, Nigeria ^6^Lagos State Health Management Agency, Lagos, Nigeria
Name and contact information for the trial sponsor {5b}	Dr. Olusegun Ogboye, BSc, MBBS, MPH Permanent Secretary, Lagos State Ministry of Health, Lagos, Nigeria. email: olusegun.ogboye@lagosstate.gov.ng
Role of sponsor {5c}	The study sponsor and funder do not have a role in the design; collection, management, analysis, and interpretation of data; writing of the report; and the decision to submit the this or other manuscripts for publication.

## Introduction

### Background and rationale {6a}

#### Study rationale

The severe acute respiratory syndrome coronavirus 2 (SARS-CoV-2) causes the coronavirus disease 2019 (COVID-19), and it was first detected in Wuhan, China, in December 2019 [1]. COVID-19 disease was recognized as a pandemic by the World Health Organization (WHO) less than 3 months after on the 11th of March 2020 [2]. By July 1, 2020, more than 10,357,662 cases of COVID-19 had been reported, with about 508,055 deaths globally [3, 4]. COVID-19 outbreak forecasts and mathematical models suggest that the size of the population affected will continue to rise [5] in the coming months.

The virus is typically spread from one person to another via respiratory droplets produced during speech, coughing and sneezing [6]. Primarily, it spreads when people are in close contact, but it may also spread when one touches a contaminated surface and then their eyes, nose or mouth. It is most contagious when people are symptomatic, although spread may be possible before symptoms appear. The time between exposure and symptom onset is typically around 5 days but may range from 2 to 14 days. Fever and chills and dry cough, as well as shortness of breath, are commonly reported symptoms [1]. Complications may include pneumonia and acute respiratory distress syndrome. A WHO investigative report indicates that age over 60 years, diabetes mellitus, cardiovascular disease, chronic respiratory disease and cancer are associated with the highest risk of severe disease and death [1].

In Nigeria, a total of 26,484 cases and 603 deaths had been reported as of 1 July 2020 with Lagos alone accounting for about half of the cases [7]. The high proportion of cases contributed by Lagos State is likely due to the fact that it is the commercial capital with a large number of international travellers and has COVID-19 testing facilities and a very large population compared to other states.

Coronavirus is a type of single-stranded positive-strand RNA virus with an envelope [8]. Currently, there is no specific therapy for coronavirus infections. Only a limited number of treatment studies have been conducted because most human coronavirus strains cause self-limiting disease and care is supportive. However, after the severe acute respiratory syndrome (SARS) coronavirus was identified in 2002 and caused a large global outbreak, there was an increased interest in the development of specific therapeutic agents. SARS-CoV case-patients were treated with corticosteroids, chloroquine phosphate, hydroxychloroquine, convalescent plasma, and lopinavir or ritonavir, and, except for ribavirin, many of these agents have in vitro preclinical data that support their efficacy [9–19]. Since the SARS outbreak, new therapeutic agents targeting viral entry proteins, proteases, polymerases and methyltransferases have been tested; however, none of them has been shown to be efficacious in clinical trials [20–22].

There are reports about the potential value of a number of drugs [9–12, 23]. Of the various therapeutic interventions that have been suggested as being potentially effective, chloroquine stands out as an attractive option for the treatment of COVID-19 in Nigeria, if found to be efficacious. It has been used extensively for the treatment of malaria in Nigeria and is low-cost. Clinically, it has been reported that chloroquine improves outcomes in patients with COVID-19 [24–26]. However, robust clinical trials are still required to prove their effectiveness and safety in the treatment of COVID-19 due to limitations in the design of reported clinical studies. Furthermore, there are recent reports of chloroquine poisoning following announcements of its potential effectiveness in the treatment of COVID-19 [27]. This compelled a notice by the Nigeria Centre for Disease Control (NCDC) publicly stating that chloroquine is not an approved treatment for COVID-19 [28].

Chloroquine has been used clinically since 1944, primarily for the prevention and treatment of malaria [29]. More recently, it has been shown to have antiviral activity against HIV-1, avian influenza H5N1, HCoV-229E, hepatitis B and herpes simplex type 1 viruses [29–32]. Chloroquine’s anti-SARS-CoV activity involves a variety of mechanisms that inhibit virus replication such as suppressing phosphatidylinositol binding clathrin assembly protein (PICALM), inhibiting endosomal acidification required for virus/cell fusion and reducing terminal glycosylation of angiotensin-converting enzyme 2 (ACE2) receptors [31, 33, 34]. Chloroquine also suppresses tumour necrosis factor alpha and interleukin 6, which mediate the inflammatory complications of viral infection [29].

The inhibition of infection at the cellular level has been shown to occur at a 50% inhibitory concentration (*IC50*) of 8.8 ± 1.2 μM for SARS-CoV [31] and 50% maximal effective concentration (*EC50*) of 1.13 μM for SARS-CoV-2 [9]. Furthermore, an *EC90* of 6.9 μM was achieved for SAR-CoV-2 [9]. These findings are significant because they suggest that therapeutic doses needed for effective anti-SARS-CoV-2 activity are similar to doses used in the treatment of malaria, which is endemic in Nigeria, and rheumatoid arthritis [31, 35]. Hydroxychloroquine has not been studied to the same extent, but it appears to have more potent in vitro efficacy [36]. These concentrations of chloroquine are significantly lower than the 50% cytotoxic concentration (*CC50*) which ranged from greater than 100 μm to 261.3 ± 14.5 μm with selectivity index from 30 to more than 88 [9, 31].

Despite the promising results from in vitro studies, chloroquine has not been shown conclusively to be efficacious in the treatment of COVID-19. However, it is now part of the recommendations for treatment in several countries while awaiting results of planned and on-going clinical trials [23, 36]. These recommendations have been informed by two main sources—an expert consensus on chloroquine phosphate for treatment of COVID-19 from China and a clinical study undertaken in France [26, 37]. These and one other small study reported from China suggest that chloroquine and hydroxychloroquine prevent the worsening of pneumonia, facilitate the clearance of the virus and shorten the duration of COVID-19 [24–26, 37].

There are very few clinical studies on the efficacy of chloroquine phosphate or hydroxychloroquine sulphate. In the main study that has generated expectations of a cure, hydroxychloroquine appeared to lead to a rapid decline in nasopharyngeal viral load with negative viral cultures in almost all patients at day 5 [26]. The duration of hospital admission was also short averaging 5 days. Although impressive, this evidence is considered weak [23], due to the methodological design of the study which had a small sample size and no control group.

Similar to chloroquine phosphate and hydroxychloroquine sulphate, there are few clinical studies investigating the effect of lopinavir/ritonavir in the treatment of COVID-19 disease [38]. The combination of antiviral serum protease inhibitors lopinavir and ritonavir has been touted as a potential therapy for COVID-19 disease. Both drugs have historically been used to manage human immunodeficiency disease type 1 (HIV-1) and found to have an in vitro inhibitory effect on the SARS-CoV virus [11, 23, 39, 40]. The antivirals work synchronously with ritonavir which inhibits the enzyme cytochrome P450 and by effect increases the plasma half-life of lopinavir [11].

Cao et al. [38] conducted an open-label randomized controlled trial in hospitalized patients using lopinavir/ritonavir in combination with standard care therapy. The study found that lopinavir/ritonavir in combination with standard therapy did not provide a clinical benefit or reduce mortality compared to standard therapy alone. However, the study was not blinded, meaning the knowledge of the assigned treatment could have biased the findings of the outcome assessors.

The relatively high morbidity associated with COVID-19 and potentially inappropriate use of publicized remedies means that identifying effective medications that can reduce the duration and severity of illness is a high priority globally. The World Health Organization is taking steps towards a multi-country clinical trial of potential therapeutic agents [41]. This study is being undertaken as part of global efforts to identify effective specific therapeutic agents for COVID-19.

Broadly, this study, is asking three different questions, namely (i) does chloroquine phosphate safely improve patient outcome when added to the standard treatment for COVID-19 compared to the addition of placebo?; (ii) does hydroxychloroquine sulphate safely improve patient outcome when added to the standard treatment for COVID-19 compared to the addition of placebo?; and (iii) does lopinavir/ritonavir safely improve patient outcome when added to the standard treatment for COVID-19 compared to the addition of placebo?

#### Importance of the Lagos COVID-19 Treatment Trial

Early identification of effective remedies that can shorten the duration and severity of illness is critical for Lagos State and Nigeria. The COVID-19 pandemic places an enormous burden on the Nigerian health system which already faces significant resource constraints. There is a limited number of hospital beds, especially, for infectious diseases requiring isolation and severely ill patients who need high dependency or intensive care. The availability of such treatment would reduce morbidity and mortality and increase the availability of hospital beds, thereby, enabling management of the epidemic without compromising care for patients with other diseases. Effective treatment may also lead to an earlier resolution of the pandemic.

Testing these drugs in a robust trial is important because it would help determine conclusively whether or not they are efficacious. If they are, then the scenario above applies, while if they are not then efforts can be redirected to examine other potential remedies and resources would not be spent on ineffective therapies.

## Objectives {7}

1. To evaluate the clinical efficacy of chloroquine phosphate, hydroxychloroquine sulphate and lopinavir/ritonavir added on to standard therapy relative to standard therapy alone for treatment of individuals with laboratory-confirmed COVID-19
  1.1. To evaluate the clinical efficacy of chloroquine phosphate added on to standard therapy relative to standard therapy alone for treatment of individuals with laboratory-confirmed COVID-19
  1.2. To evaluate the clinical efficacy of hydroxychloroquine sulphate added on to standard therapy relative to standard therapy alone for treatment of individuals with laboratory-confirmed COVID-19
  1.3. To evaluate the clinical efficacy of lopinavir/ritonavir added on to standard therapy relative to standard therapy alone for treatment of individuals with laboratory-confirmed COVID-19

The secondary objectives are:

To evaluate the effect of chloroquine phosphate, hydroxychloroquine sulphate and lopinavir/ritonavir added on to standard therapy compared to standard therapy on the rate of progression/resolution of COVID-19
  1.1. To evaluate the effect of chloroquine phosphate added on to standard therapy compared to standard therapy on the rate of progression/resolution of COVID-19
  1.2. To evaluate the effect of hydroxychloroquine sulphate added on to standard therapy compared to standard therapy on the rate of progression/resolution of COVID-19
  1.3. To evaluate the effect of lopinavir/ritonavir added on to standard therapy compared to standard therapy on the rate of progression/resolution of COVID-19
2. To evaluate the safety of chloroquine phosphate, hydroxychloroquine sulphate and lopinavir/ritonavir added on to standard therapy compared to standard therapy in the treatment of individuals with laboratory-confirmed COVID-19
  2.1. To evaluate the safety of chloroquine phosphate added on to standard therapy compared to standard therapy in the treatment of individuals with laboratory-confirmed COVID-19
  2.2. To evaluate the safety of hydroxychloroquine sulphate added on to standard therapy compared to standard therapy in the treatment of individuals with laboratory-confirmed COVID-19
  2.3. To evaluate the safety of lopinavir/ritonavir added on to standard therapy compared to standard therapy in the treatment of individuals with laboratory-confirmed COVID-19

## Trial design {8}

This is a multi-centre, randomized, double-blind, placebo-controlled multi-arm clinical trial with a parallel group and superiority design. There are three treatment arms and one control arm with equal distribution of participants between the arms. If the intervention drug for any arm is not available, then participants will be allocated equally to each of the available arms.

## Methods: participants, interventions and outcomes

### Study setting {9}

This trial will take place at health facilities designated for COVID-19 patients in Lagos State, Nigeria. The LACTT trial will be conducted at three of the health facilities designated for COVID-19 patients in Lagos State, Nigeria. The centres are Lagos Mainland Infectious Disease Hospital, Yaba; the Eti-Osa COVID-19 Isolation and Treatment Centre; and the Onikan COVID-19 Isolation and Treatment Centre Lagos State. The three centres manage patients domiciled in Lagos State, based on a similar state-wide protocol which aligns with the Nigeria Centre for Disease Control guideline for testing and management of COVID-19 disease. The proposed sample size distribution across the three centres is 200 participants per centre.

### Eligibility criteria {10}

#### Inclusion criteria

In order to be eligible to participate in this study, a patient must meet all of the following criteria: (1) provide written informed consent prior to initiation of any study procedures; (2) understand and agree to comply with planned study procedures; (3) agree to the collection of oropharyngeal swabs and venous blood per protocol; (4) be male or non-pregnant female adult ≥18 years of age at the time of enrolment; and (5) have laboratory-confirmed SARS-CoV-2 infection as determined by RT-PCR in any specimen no more than 72 h prior to randomization. Both asymptomatic and symptomatic cases (mild/moderate/severe) are eligible for enrolment.

#### Exclusion criteria

An individual who meets any of the following criteria will be excluded from participation in this study:

1. ALT/AST > 5 times the upper limit of normal
2. Stage 4 severe chronic kidney disease or requiring dialysis (i.e. eGFR < 30)
3. Pregnant or breast feeding
4. Anticipated transfer to another facility which is not a study site within 72 h
5. Participants with known haematological diseases, e.g. G6PD deficiency
6. Participants with chronic liver and kidney disease and reaching end-stage
7. Participants with arrhythmia and chronic heart disease
8. Participants known to have retinal disease or hearing loss
9. Participants known to have a mental illness
10. Skin disorders (including rash, dermatitis, psoriasis)
11. Allergy to any study medication (4-aminoquinoline or lopinavir/ritonavir)

#### Lifestyle consideration

Participants will be advised to refrain from alcohol and tobacco use during the period of study.

### Who will take informed consent? {26a}

Informed consent will be taken by trained clinical research assistants (CRAs). Discussions of essential information about the research will include the study’s purpose, duration, experimental procedures, alternatives, risks and benefits, and subjects will have the opportunity to ask questions and have them answered. There will be interpreters at the study sites for potential participants who cannot communicate in English.

### Additional consent provisions for collection and use of participant data and biological specimens {26b}

Potential participants will be asked for consent to collect additional blood by CRAs, the use of residual specimens and samples for secondary research. Extra blood will be drawn for secondary research by CRAs during designated days when study blood samples are obtained.

The stored samples will be labelled with codes to maintain confidentiality. Research with identifiable samples and data may occur as needed; however, subject confidentiality will be maintained as described for this protocol and with HREC approval. Samples designated for secondary research use may be used for understanding the SARS-CoV-2 infection, the immune response to this infection, and the effect of therapeutics on these factors. Samples will not be sold. Although the results of any future research may be patentable or have commercial profit, participants will have no legal or financial interest in any commercial development resulting from any future research.

There are no direct benefits to the participant for extra specimens collected or from the secondary research. No results from secondary research will be entered into the participant’s medical record. Incidental findings will not be shared with the participant, including medically actionable incidental findings, unless required by law. Participants may withdraw permission to use samples for secondary use at any time. They will need to contact the study site and the samples will be removed from the study repository after this study is completed and documentation will be completed that outlines the reason for withdrawal of permission for secondary use of samples.

## Explanation for the choice of comparators {6b}

The comparator for this study is the standard of care at each of the study sites.

The standard supportive for adults with COVID-19 are as stated below (Table 2).

**Table 2:** Standard of care for COVID-19 at study sites

Standard supportive therapy	Explanation
Supplemental oxygen therapy immediately to patients with severe acute respiratory infection (SARI) and respiratory distress, hypoxaemia or shock.	• Commence oxygen therapy at 5 L/min and titrate flow rates to reach target SpO_2_ ≥ 90% in non-pregnant adults and SpO_2_ ≥ 92–95% in pregnant patients. • Children with emergency signs, such as breathing difficulty, obstruction or apnea, severe respiratory distress, central cyanosis, shock, coma or convulsions, should receive oxygen therapy during resuscitation to target SpO_2_ ≥ 94%; otherwise, the target SpO_2_ is ≥ 90%. • Wards and outpatients where patients with SARI are cared for are equipped with pulse oximeters, functioning oxygen systems and disposable, single-use, oxygen-delivering interfaces (nasal cannula, simple face mask and mask with reservoir bag). • Contact precautions will be taken when handling contaminated oxygen interfaces of patients with SARS-COV-2 infection.
Conservative fluid management in patients with SARI when there is no evidence of shock.	• Patients with SARI will be treated cautiously with intravenous fluids, because aggressive fluid resuscitation may worsen oxygenation.
Empiric antimicrobials to treat all likely pathogens causing SARI. Antimicrobials (Augmentin 650mg tablets 12hhrly for 24h or Augmentin IV 1g q 24 h) within 1 h of initial patient assessment for patients with evidence of URTI or sepsis.	• The clinicians will administer appropriate empiric antimicrobials within 1 h of identification of sepsis or URTI. • Empiric antibiotic treatment will be based on the clinical diagnosis (community-acquired pneumonia, healthcare-associated pneumonia [if infection was acquired in healthcare setting] or sepsis). Empiric therapy will be de-escalated on the basis of microbiology results and clinical judgment.
• Zinc sulphate tablets 100mg daily • Calcium tablets 300mg daily • Vitamin C tablets 1g daily • Vitamin D tablets 50mcg daily	• Given to all individuals with COVID-19

## Intervention description {11a}

The dose and route of administration of the trial drugs are fixed. The medications will be taken with food. Missed doses for the day will not be made up. All intervention medications will be administered with the standard of care.

### Arm 1

In the chloroquine phosphate arm of the study, participants will receive chloroquine phosphate two 250mg tablets orally twice a day (12h/24h) for 7 days, two hydroxychloroquine placebo tablets orally twice a day (12h/24h) for 7 days and two lopinavir/ritonavir placebo tablets orally twice a day (12h/24h) for 14 days.

### Arm 2

In the hydroxychloroquine arm, participants will receive hydroxychloroquine sulphate two 200mg tablets orally twice a day (12h/24h) for 7 days, two chloroquine placebo tablets orally twice a day (12h/24h) for 7 days and two lopinavir/ritonavir placebo tablets orally twice a day (12h/24h) for 14 days.

### Arm 3

In the lopinavir/ritonavir arm, participants will receive two tablets of lopinavir 200mg/ritonavir 50mg combination tablet orally twice a day (12h/24h) for 14 days, two chloroquine placebo tablets orally twice a day (12h/24h) for 7 days and two hydroxychloroquine placebo tablets orally twice a day (12h/24h) for 7 days.

### Arm 4

In the control arm of the study, participants will receive two chloroquine placebo tablets orally twice a day (12h/24h) for 7 days, two hydroxychloroquine placebo tablets orally twice a day (12h/24h) for 7 days and two lopinavir/ritonavir placebo tablets orally twice a day (12h/24h) for 14 days.

If any of the treatment arms is not available, the placebo for the relevant drug will not be administered to participants in the other arms.

## Criteria for discontinuing or modifying allocated interventions {11b}

If the estimated creatinine clearance decreases by more than ≥ 50% from baseline, the study interventions (oral chloroquine phosphate, hydroxychloroquine sulphate and lopinavir/ritonavir) will not be given. The interventions may be resumed when the estimated creatinine clearance returns to baseline. If the liver function tests (ALT and/or AST) increase to > 2 times upper limits of normal, the dose of chloroquine phosphate, hydroxychloroquine sulphate and lopinavir/ritonavir will not be administered. Dosing may be resumed when the ALT and/or AST returns to baseline. Dosing may be given later the same day. If a day’s dosing is missed, it will not be made up.

If any of the following occur, the dose of chloroquine phosphate or hydroxychloroquine sulphate or lopinavir/ritonavir will be stopped and will not be restarted:

- ALT ≥3 × upper limits of normal and bilirubin ≥2 × upper limits of normal
- ALT and/or AST increases to > 5 times upper limits of normal

## Strategies to improve adherence to interventions {11c}

Participants will be directly observed taking their medications by clinical nurses and CRAs at the study sites to encourage and monitor adherence.

## Relevant concomitant care permitted or prohibited during the trial {11d}

Non-supportive therapy prior to enrolment is permitted. Concomitant therapy for co-morbidities such as hypertension, diabetes, rheumatoid arthritis etc. will be permitted and documented. In addition, the list of participants’ medications will be assessed (from medical history) from at least 30 days prior to enrolment to the 15th day after enrolment.

## Provisions for post-trial care {30}

Study personnel will try to reduce, control and treat any harm from trial participation. Immediate medical treatment will be provided at Lagos Mainland Hospital Yaba. As needed, referrals to appropriate healthcare facilities will be provided to the subject. The site study physician will then determine if an injury occurred as a direct result of the tests or treatments that are done for this trial.

If it is determined by the site study physician that an injury occurred to a subject as a direct result of the tests or treatments that are done for this trial, then referrals to appropriate healthcare facilities will be provided to the subject. In case of SAEs or other research-related injuries, the full cost of treatment and ensuing cost associated with any associated complications will be borne by study sponsors.

## Outcomes {12}

COVID-19 is a new disease and our understanding of it is evolving. To align this study with other international studies, the objectives, endpoints and some other aspects of the WHO’s Master Protocol [42] have been adopted as appropriate with modifications to suite the Lagos context. There are three objectives—one primary and two secondary objectives. Each objective has three sub-objectives—one for comparing chloroquine phosphate added on to standard therapy and standard therapy alone; one for comparing hydroxychloroquine sulphate added on to standard therapy and standard therapy alone; and one for comparing lopinavir/ritonavir added on to standard therapy and standard therapy alone.

A special WHO committee developed the ordinal scale that will be used in this study for the primary endpoint [43]. This study uses a combined endpoint (based on the WHO 7-point ordinal scale) of both “clinical status and mortality” at day 15. Hence, superiority can be demonstrated when either arm shows a significant effect on the variables for clinical status OR mortality by day 15. This endpoint is particularly useful in our population given the low mortality rate, and a combined endpoint would increase the study power. The secondary endpoints will enable evaluation of the effect of the trial medications on the rate of change in the clinical condition of COVID-19 patients, as well as the safety of the trial medications (Table 3).

**Table 3:** LACTT objectives and endpoints

**Primary objectives**	**Primary endpoints**
1. To evaluate the clinical efficacy of chloroquine phosphate, hydroxychloroquine sulphate and lopinavir/ritonavir added on to standard therapy relative to standard therapy alone for treatment of individuals with laboratory-confirmed COVID-19 1.1 To evaluate the clinical efficacy of chloroquine phosphate added on to standard therapy relative to standard therapy alone for treatment of individuals with laboratory-confirmed COVID-19 1.2 To evaluate the clinical efficacy of hydroxychloroquine sulphate added on to standard therapy relative to standard therapy alone for treatment of individuals with laboratory-confirmed COVID-19 1.3 To evaluate the clinical efficacy of lopinavir/ritonavir added on to standard therapy relative to standard therapy alone for treatment of individuals with laboratory-confirmed COVID-19	*Seven-point ordinal scale assessed on day 15* 1. Not hospitalized, no limitations on activities 2. Not hospitalized, limitation on activities 3. Hospitalized, not requiring supplemental oxygen 4. Hospitalized, requiring supplemental oxygen 5. Hospitalized, on non-invasive ventilation or high flow oxygen devices 6. Hospitalized, on invasive mechanical ventilation 7. Death
**Secondary objectives**	**Secondary endpoints**
1. To evaluate the effect of chloroquine phosphate, hydroxychloroquine sulphate and lopinavir/ritonavir added on to standard therapy compared to standard therapy on the rate of progression/resolution of COVID-19 1.1 To evaluate the effect of chloroquine phosphate added on to standard therapy compared to standard therapy on the rate of progression/resolution of COVID-19 1.2 To evaluate the effect of hydroxychloroquine sulphate added on to standard therapy compared to standard therapy on the rate of progression/resolution of COVID-19 1.3 To evaluate the effect of lopinavir/ritonavir added on to standard therapy compared to standard therapy on the rate of progression/resolution of COVID-19	1. Clinical status on the 7-point ordinal scale on days 3, 5, 8, 11 and 29 2. National Early Warning Score assessed daily while hospitalized and on day 15 3. Duration of supplemental oxygen 4. Duration of mechanical ventilation 5. Duration of hospitalization or isolation 6. SARS-CoV-2 clearance time based on samples taken on days 3, 5, 8, 11, 15 and 29
2. To evaluate the safety of chloroquine phosphate, hydroxychloroquine sulphate and lopinavir/ritonavir added on to standard therapy compared to standard therapy in the treatment of individuals with laboratory-confirmed COVID-19 2.1 To evaluate the safety of chloroquine phosphate added on to standard therapy compared to standard therapy in the treatment of individuals with laboratory-confirmed COVID-19 2.2 To evaluate the safety of hydroxychloroquine sulphate added on to standard therapy compared to standard therapy in the treatment of individuals with laboratory-confirmed COVID-19 2.3 To evaluate the safety of lopinavir/ritonavir added on to standard therapy compared to standard therapy in the treatment of individuals with laboratory-confirmed COVID-19	1. Cumulative incidence of severe adverse events up to and including day 29 2. Changes in laboratory safety indices assessed on days 1, 8 and 15—haemoglobin concentration, platelet count, white blood cell count, glucose, creatinine, total bilirubin, ALT and AST

## Efficacy assessments

The schedule of assessments (SOA) in Table 4 outlines the baseline and follow-up assessments that will be done.

**Table 4:** Schedule of assessments

Study period	Screening	Baseline	Follow-up days											
Day ± window	−1 or 1	1	2	3	4	5	6	7	8	9	10	11–14	15 ± 2	29 ± 3
1. Eligibility														
Informed consent	X													
Demographics and medical history	X													
Lung auscultation	X													
Chest radiography	X													
Confirm SARS-CoV-2 results^1^	X													
2. Study interventions														
Randomization		X												
Administration of study drugs^2^		X	X	X	X	X	X	X	X	X	X	X		
3. Study procedures														
Vital signs		X	X	X	X	X	X	X	X	X	X	X	X	
Clinical assessment^3^		X	X	X	X	X	X	X	X	X	X	X	X	
Review treatment/management		X	X	X	X	X	X	X	X	X	X	X	X	X
ECG for safety		X		X		X			X			X	X	X
Adverse event evaluation		X	X	X	X	X	X	X	X	X	X	X	X	X
4. Laboratory tests for safety														
Full blood count, liver function tests, creatinine^4^		X							X				X	
Pregnancy test for women of reproductive age	X													
5. SARS-CoV-2 testing														
PCR for SARS-CoV-2		X		X		X			X			X	X	X
6. Sample for future testing														
Oropharyngeal swabs^5^		X							X				X	
Blood/serum^5^		X							X				X	

^1^Time frame for test results: Results of tests done within 72 h of enrolment will be used

^2^Treatment duration: If the patient is still on admission after day 14, standard treatment will continue in all arms of the study while study medications stop on day 7 for chloroquine and hydroxychloroquine and on day 14 for lopinavir/ritonavir

^3^Clinical data to be collected: See the “Efficacy assessments” section for details

^4^Laboratory tests for safety: Blood—haemoglobin, white cell count, platelets, glucose, creatinine, bilirubin, alanine aminotransferase, aspartate aminotransferase

^5^Samples for future research: To be used when additional SARS-CoV-2 tests become available

### Measures of clinical status

On each study day while hospitalized or in isolation, the following measure of clinical status will be assessed:

- Symptoms
- Vital signs
- Oxygen requirement
- Non-invasive mechanical ventilation (via mask)
- Mechanical ventilator requirement (via an endotracheal tube or tracheostomy tube)

### Ordinal scale

The ordinal scale is an assessment of the clinical status at the first assessment of a given study day. Each day, the worse score for the previous day will be recorded, i.e. on day 3, the day 2 score is obtained and recorded as day 2. The scale is as follows:

1. Not hospitalized, no limitations on activities
2. Not hospitalized, limitation on activities
3. Hospitalized, not requiring supplemental oxygen
4. Hospitalized, requiring supplemental oxygen
5. Hospitalized, on non-invasive ventilation or high flow oxygen devices
6. Hospitalized, on invasive mechanical ventilation
7. Death

### NEW score

The NEW score is based on 7 clinical parameters (see Table 5) and is being used as an efficacy measure. This will be evaluated at the first assessment of a given study day. These parameters can be obtained from the hospital chart using the last measurement prior to the time of assessment. This is recorded for the day obtained, i.e. on day 4, the day 3 score is obtained and recorded as day 3.

**Table 5:** NEW score

Physiologic parameters	Score						
	3	2	1	0	1	2	3
Respiratory rate	≤8		9–11	12–20		21–24	≥25
Oxygen saturation	≤91	92–93	94–95	≥96			
Supplemental oxygen		Yes		No			
Temperature	≤35.0		35.1–36.0	36.1–38.0	38.1–39.0	≥39.1	
Systolic BP	≤90	91–100	101–110	111–219			≥220
Heart rate	≤40		41–50	51–90	91–110	111–130	≥131
Level of consciousness*				A			VPU

*Level of consciousness: alert (A), arousable only to voice (V) or pain (P), and unresponsive (U)

### Further assessments

Oropharyngeal swabs and blood/serum samples will be collected on days 1, 8 and 15 (while hospitalized or in isolation) and stored. Tests for viral shedding and antiviral antibodies are being developed and will be used to test the stored sample when they become available.

## Safety assessments

Study procedures are specified in the SOA. A study physician will be responsible for all trial-related medical decisions.

- Physical examination
  - ◦ A symptom-directed (targeted) physical examination will be performed to evaluate for any possible adverse event.
- Clinical laboratory evaluations
  - ◦ Fasting is not required before collection of laboratory samples.
  - ◦ Blood will be collected at the time points indicated in the SOA. Clinical laboratory parameters include WBC, Hb, PLT, Cr, glucose, total bilirubin, AST and ALT.

If a physiologic parameter, e.g. vital signs, or laboratory value is outside of the protocol-specified range (Table 6), then the measurement may be repeated once if, in the judgment of the investigator, the abnormality is the result of an acute, short-term, rapidly reversible condition or was an error. A physiologic parameter may also be repeated if there is a technical problem with the measurement caused by malfunctioning, or an inappropriate measuring device (i.e. inappropriate-sized BP cuff).

**Table 6:** Normal reference ranges for vital signs and safety assessments

Parameter	Reference range
Temperature	36.1–37.2°C
Pulse rate	60–100 beats/min
Respiratory rate	12–16 cycles/min
Systolic blood pressure	90–130 mmHg
Diastolic blood pressure	70–90 mmHg
Haemoglobin	12–17.5 g/dl
White blood cell count	4.00–11.0 × 109/l
Platelet count	150,000–450,000/μl
Creatinine	0.5–1.1mg/dl
Random glucose	76–150mg/dl
Total bilirubin	0–1.0mg/dl
ALT	0–40u/l
AST	0–37i.u/l

## Participant timeline {13}

**Fig. 1:**
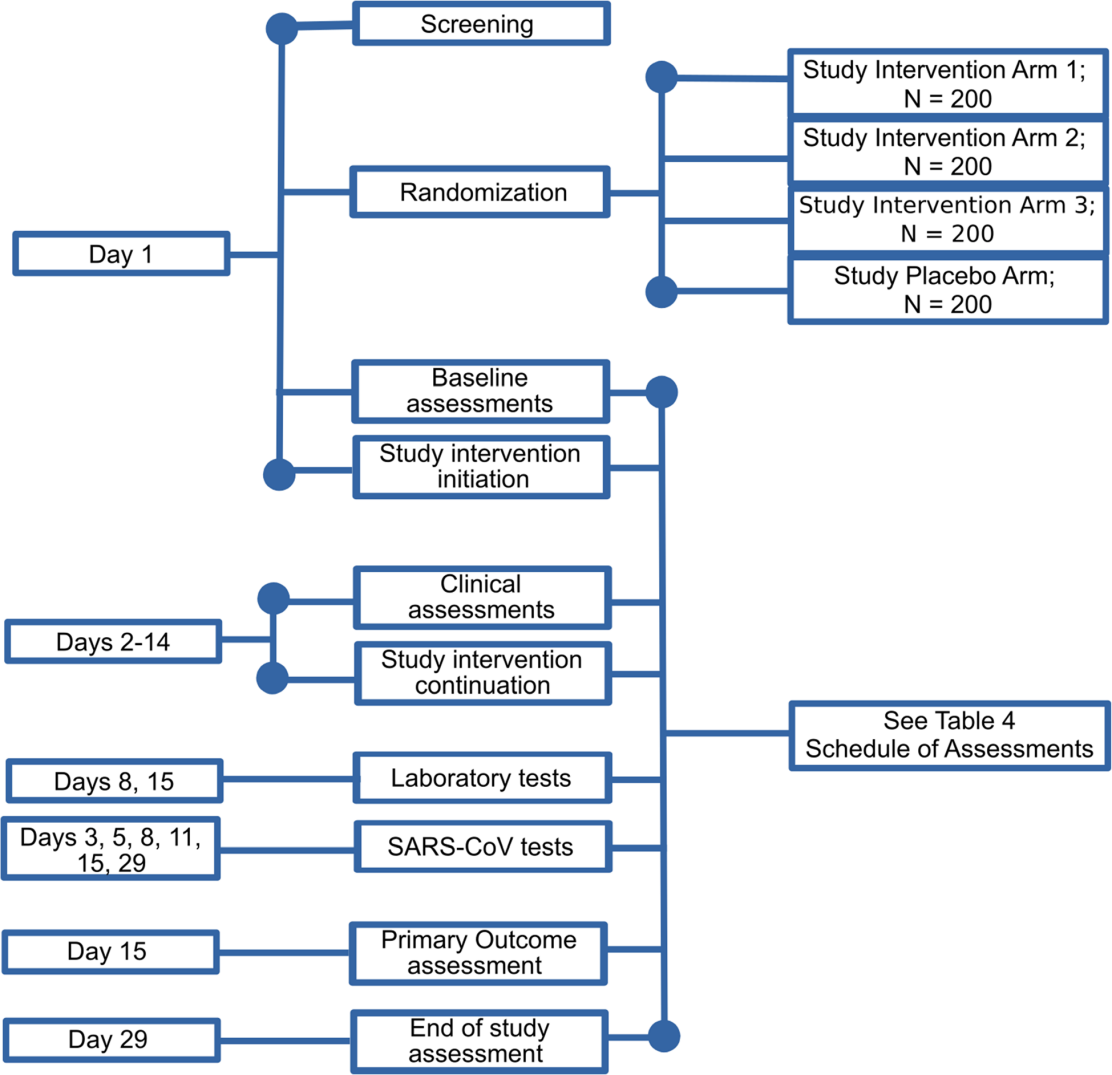
Schematic diagram showing the timeline for key processes in LACTT

The participant timeline is shown in Fig. 1.

## Sample size {14}

The sample size of 200 per arm has been calculated assuming an odds ratio (*OR*) of 2, 85% power and a 2-tailed test at level *α* = 0.05 and allowing for approximately 10% of participants to be lost to follow-up. The odds ratio represents the odds of improvement in the ordinal scale for treatment relative to control.

## Recruitment {15}

It is anticipated that patients with COVID-19 will present to the participating hospitals which are designated COVID-19 treatment centres. Therefore, no other efforts to recruit potential subjects are needed.

## Assignment of interventions: allocation

### Sequence generation {16a}

Prior to study initiation, a randomization list will be generated (using the statistical programming language R, version 3.6.3 to enable random assignment to each of the trial arms at each of the study sites using an equal allocation ratio (for instance 1:1:1:1 for four arms)). If arms are added to or dropped from the trial, randomization will proceed with an equal probability of assignment to each of the remaining arms.

Randomization will be stratified by site and at each site by severity of illness at enrollment yielding two strata: (1) asymptomatic (no clinical symptoms or signs) and (2) symptomatic (presence of clinical symptoms and/ or signs). The R code used to generate the randomization lists will store the seed so that the generated lists are reproducible.

The randomization list will be generated using permuted block randomization with two block sizes, with the larger block size chosen so that it is not a multiple of the smaller block. Using permuted block randomization rather than simple randomization will avoid the possibility of long periods of time during which participants are not allocated to one or more arms of the study—a scenario which could introduce bias to the study due to time effects. Two block sizes would make it more difficult for clinicians (who will be unaware of the block sizes) to guess any part of the allocation sequence, thus, reducing the potential for the introduction of selection bias by well-meaning staff members at study sites.

### Concealment mechanism {16b}

Eligible patients will be allocated to receive medication in individually numbered packs, according to the sequential order of the randomization list. These will be packaged by an unblinded team at the Lagos State central pharmacy lead by the clinical research pharmacist. These personnel are otherwise not involved in the management/care and assessment of the study participants. Neither the names of the investigational drugs nor the arm of the study will be included in the medication packs.

After assignment to interventions, trial participants, care providers and outcome assessors will remain blinded through the use of placebo that will have the same physical formulation, route of administration and dosing schedule.

### Implementation {16c}

The allocation sequence will be generated by a member of the trial team who will be based away from the trial sites and will not be involved in care of the participants, outcome assessment or management/analysis of unblinded data.

Clinical research assistants will enrol participants and assign intervention medication packs in sequential order of the randomization list and stratification.

## Assignment of interventions: blinding

### Who will be blinded {17a}

Trial participants, the clinical care team, research team, outcome assessors, data management personnel and data analysts will be blinded.

### Procedure for unblinding if needed {17b}

Individual envelopes containing the arm of the study and trial drugs enclosed in each numbered medication pack will be prepared for emergency unmasking in cases of serious adverse events (SAEs) and serious and unexpected suspected adverse reactions (SUSARs).

To unblind a participant, the study physician at the concerned study site will send the completed unblinding request form via email or manually to the clinical oversight lead at the study site. The study site pharmacist will be notified in the communication as well. The clinical oversight lead will notify the principal investigator and authorize unblinding.

Once the authorization request is approved, the site pharmacist will proceed to release the coded envelope for the research participant concerned. The site pharmacist will also file a copy of the unblinding request form in the pharmacy records for the study. The site pharmacist will ensure that the treatment assignment of the research participant concerned is shared with the study physician for the study site alone who would in turn take the necessary actions for the management of the patient and reporting adverse events and SUSARs.

## Data collection and management

### Plans for assessment and collection of outcomes {18a}

Participant data will be collected using case reporting forms (on mobile tablets with REDCap®) to be completed by trained clinical research assistants at baseline all through day 15 and then day 29. The use of REDCap® with periodic data reviews by a data management and auditing team will help the research team maintain data quality.

The schedule of assessments is shown in Table 4 and illustrated in Fig. 1. See Supplements for case reporting forms.

#### SOC available in each participating study site

Data on the standard of care available and provided to each participant will be summarized for each study site and the entire study population.

### Plans to promote participant retention and complete follow-up {18b}

Research participants in the treatment and control arms will be encouraged by the research team and given access to COVID-19 educational resources during the study. In addition, participants will receive communication from the research welfare team to attend to any concerns or questions participants may have during the study.

For participants who deviate or discontinue from the protocol, all outcome data relevant to the study prior to deviation or discontinuation from the study protocol will be collected.

### Data management {19}

The site investigator is responsible for assuring that the data collected is complete, accurate and recorded in a timely manner. Study data will be entered into REDCap® using tablets. Source documentation (the point of initial recording of information) will be signed and dated by the person recording and/or reviewing the data and will support the data transferred to REDCap®. The data entered into REDCap® will be compared with source documentation on a regular periodic bases as part of data quality assurance.

Due to the biohazard of SARS-COV-2 virus contamination, any original written source documents created at the bedside (i.e. in the ‘red zone’) will be treated as potentially infectious unless an effective means of decontamination can be instituted. Where possible, photographs, digital scans or other electronic data capture methods may be obtained instead of transporting the original written source documents from the ‘red zone’. Interview of subjects will be used for obtaining medical history.

### Confidentiality {27}

All records will be kept confidential to the extent provided by Lagos State University Teaching Hospital Health Research Ethics Committee (LASUTH HREC) regulations. The study monitors and other authorized representatives of the Sponsor may inspect all documents and records required to be maintained by the investigator, including but not limited to, medical records. Records will be kept locked and all computer entry will be done with coded numbers only. Clinical information will not be released without written permission of the subject, except as necessary for monitoring by LASUTH HREC.

### Plans for collection, laboratory evaluation and storage of biological specimens for genetic or molecular analysis in this trial/future use {33}

Any use of the sample or data for secondary research purposes, however, will be presented in a separate protocol and require separate HREC approval. Each sample will be labelled only with participant ID to protect subject confidentiality.

Each participant can withdraw consent at any time by notifying the study physicians or nurses in writing. If the subject subsequently changes his/her decision, the samples will be destroyed if the samples have not been used for research or released for a specific research project.

## Statistical methods

### Statistical methods for primary and secondary outcomes {20a}

The statistical methods are based mainly on the WHO Master Protocol [42], as outlined below.

#### Statistical methods for analyzing the primary outcome

The primary hypothesis is that the clinical status measured by a 7-point ordinal scale for each of the investigational arms (1, 2, 3) will be different from that of the placebo arm.

Boschloo’s test will be used to evaluate this primary hypothesis in all randomized participants (intention-to-treat analysis). For each comparison of a given arm to control, only participants concurrently randomized to those two arms will be included. Statistical significance will be claimed if the two-sided *P*-value is less than the monitoring boundary allocating a total type I error rate of 0.05 across interim analyses. The Boschloo’s test is being used instead of Fisher’s exact test because the interim results, as well as results within subgroups, will have small sample sizes based on the multiple primary outcomes within the subgroups.

The ordinal scale will be used to estimate a proportional odds model. The primary hypothesis test will be based on a test of whether the common odds ratio for treatment is equal to one. Nonetheless, estimation and confidence intervals do require the model to be correct. Accordingly, we will evaluate model fit using a goodness-of-fit likelihood ratio test. A stratified hypothesis test to account for baseline severity of disease will be used.

The distribution of severity results will be summarized by treatment arm as percentages, and participants without final outcome data will be excluded from the analysis.

#### Statistical methods for analyzing secondary outcomes

The following analyses will be performed for the comparison of each experimental arm to the placebo arm when full enrolment has been achieved. For these analyses, the alpha level used is that of the analysis for the primary outcome when full enrolment has been achieved.

1. The Mantel-Haenszel odds ratio estimator and test statistic will be applied to the strata defined by clinical signs and symptoms (asymptomatic versus symptomatic).
2. Differences in time-to-event endpoints (e.g. time to negative SARS-CoV-2 status) by treatment will be summarized with Kaplan-Meier curves and 95% confidence bounds.
3. Change in ordinal scale at specific time points will be summarized by proportions (e.g. proportion who have a 1-, 2-, 3- or 4-point improvement or worsening).
4. Duration of event (e.g. duration of mechanical ventilation) will be summarized according to median days with quartiles.
5. Incidence data (e.g. incidence of new oxygen use) will be summarized as a percent with 95% confidence intervals.
6. Categorical data (e.g. 28-day mortality or ordinal scale by day) will be summarized according to proportions with confidence intervals on the difference or odds ratios for a binary or multiple category scale, respectively.
7. Logistic regression analyses will be undertaken to assess the effect of covariates on outcomes.

### Interim analyses {21b}

#### Interim analyses

Interim safety analyses by the Data Coordinating Centre and the DSMB will occur at approximately 25%, 50% and 75% of total enrolment. Safety analyses will evaluate serious AEs by treatment arm and test for differences using a Pocock spending function approach with a one-sided type I error rate of 0.025. This approach is less conservative than what will be used to test for early efficacy results because proving definitive harm of the experimental agents is not the focus of this study. Pocock stopping boundaries at the looks described correspond to *z*-scores of 2.37, 2.37 and 2.36. This contrasts with the *z*-score stopping boundaries for the Lan-DeMets spending function that mimics O’Brien-Fleming boundaries: 4.33, 2.96 and 2.36. The unblinded statistical team will prepare these reports for review by the DSMB.

#### Interim efficacy review

The Lan-DeMets spending function analogue of the O’Brien-Fleming boundaries will be used to monitor the primary endpoint as a guide for the DSMB for an overall two-sided type I error rate of 0.05. Interim efficacy analyses will be conducted after the DSMB has selected the primary efficacy endpoint at approximately 25%, 50% and 75% of total information.

#### Conditional power

Conditional power will be presented as an additional guide to the DSMB. Conditional power allows computation of the probability of obtaining a statistically significant result by the end of the trial given the data accumulated thus far, incorporating and assuming a hypothesized treatment effect (e.g. the treatment effect assumed for sample size determination) thereafter. If conditional power is less than 20% under the original trial assumptions, consideration will be given to stopping the trial.

#### Access to interim results and decision to terminate the trial

The unblinded statistical team will prepare the interim result reports for DSMB review and recommendations. Analyses will be presented with blinded codes for treatment arms to protect against the possibility that the DSMB report may fall into the wrong hands. A DSMB charter will further describe procedures and membership. An additional document on statistical issues related to monitoring will be provided to the DSMB prior to interim analyses. The final decision to terminate the trial or an arm of the trial based on interim findings will be made by the DSMB, chaired by an epidemiologist.

### Methods for additional analyses (e.g. subgroup analyses) {20b}

#### Evaluating safety and tolerability relative to the control arm

Safety will be evaluated by comparing the proportion of patients with at least one serious adverse event (SAE) for each study arm relative to the control arm, using a two-sided Fisher’s exact test at alpha = 0.05. Proportions of specific SAEs will be reported for each study arm. Clopper-Pearson confidence intervals for within-arm proportions will be presented.

We will tabulate the number (%) of patients with at least one SAE for each study arm. *P*-values comparing each arm to control using two-sided Fisher’s exact test at alpha = 0.05. The number (%) of patients with specific SAEs by System Organ Class (SOC) and Preferred Term (PT) will also be tabulated. *P*-values comparing each arm to control using two-sided Fisher’s exact tests at alpha = 0.05.

List of SAEs: randomized arm, days from randomization to first SAE experienced by a subject, subject ID, site, age, days from the start of study drug to SAE, SAE, severity grade, relatedness to study intervention, outcome (sort by randomized arm, subject ID, SOC and PT and, if a subject has multiple SAEs, days from randomization to SAE).

### Comparing mortality rates of investigational arms to the control arm up to 29 days after randomization

Boschloo’s test will be used to evaluate 15-day and 29-day clinical status and SARS-CoV-2 viral status in the manner proscribed for the primary and secondary analysis.

Table: Sample size, number (%) with 15-day 7-point ordinal scale clinical outcome status, number (%) missing 15-day 7-point ordinal scale clinical outcome status and 15-day SARS-CoV-2 viral status. *P*-values from Boschloo’s test comparing each arm to control.

Table: Sample size, number (%) with 29-day 7-point ordinal scale clinical outcome status, number (%) missing 29-day 7-point ordinal scale clinical outcome status and 29-day SARS-CoV-2 viral status. *P*-values from Boschloo’s test comparing each arm to control.

#### Comparing time to meeting criteria for successful discharge from the COVID-19 treatment centre between participants receiving investigational therapeutics, relative to the control arm

- Differences in median days to becoming eligible for discharge will be tested using the Wilcoxon rank-sum test, imputing deaths prior to day 28 as the worst rank, with earlier deaths having a worse rank than later deaths.
- Discharge decisions may not always be based on objective clinical data. For these reasons, time to meeting criteria for successful discharge will be analysed rather than time to actual discharge.
- We will tabulate the sample size, number (%) who became eligible for discharge within 29 days, mean, median and standard deviation of days to discharge eligibility among those for whom the endpoint is available. *P*-values for test of medians using Wilcoxon rank-sum test.
- Side-by-side plots and boxplots of time to meeting criteria for successful discharge by arm, separately for symptomatic and asymptomatic cases, will be prepared.

#### Pharmacokinetic assessments of investigational agents

In general, pharmacokinetic measurements often involve processing (e.g. centrifugation) and testing of blood specimens with techniques or equipment not routinely available or safely performed in most point-of-care laboratory set-ups. These considerations, coupled with limitations on storage, transport and analytical processing of infectious samples falling under Select Agent regulations, could limit these explorations outside the context of a high containment laboratory such as a domestic BSL-4 laboratory in Lagos State.

When possible, pharmacokinetic assessments will include summaries of mean and 95% confidence intervals for parameters such as the area under the curve (AUC) of concentration versus time, Cmax and Cmin, clearance time, volume of distribution, half-life and bioavailability.

### Methods in analysis to handle protocol non-adherence and any statistical methods to handle missing data {20c}

The analysis population will be based on intention-to-treat. No account will be taken for protocol non-adherence.

For missing data, patterns of missing data will be explored in the primary and secondary outcomes and reported. If required, multiple imputation statistical technique will be used assuming the mechanism of missing data is missing at random (MAR).

### Plans to give access to the full protocol, participant-level data and statistical code {31c}

This document is the full protocol. Anyone interested in other data or documentation should contact the corresponding author. Access to participant-level de-identified datasets and statistical codes will be made available on reasonable request to study investigators.

## Oversight and monitoring

### Composition of the coordinating centre and trial steering committee {5d}

The trial will be overseen by a Project Management Team (PMT), a Trial Committee (TC). The PMT will have a zoom meeting approximately every 2 weeks during the recruitment period and then monthly after this. The group will support any decision-making that the trial implementation team need further advice on. The TC will have a chairperson, members and trial collaborators.

### Composition of the data monitoring committee, its role and reporting structure {21a}

The Data Monitoring and Safety Board (DSMB) is responsible for safeguarding the interests of study participants, assessing the safety and efficacy of study procedures, and for monitoring the overall conduct of the study.

The DSMB is required to provide recommendations about starting, continuing and stopping the study. The DSMB is independent of the study sponsor, and members have no competing interests. In addition, the DSMB will make recommendations, as appropriate about:

- Efficacy of the study intervention
- Benefit/risk ratio of procedures and participant burden
- Selection, recruitment and retention of participants
- Adherence to protocol requirements
- Completeness, quality and analysis of measurements
- Study protocol and consent form amendments
- Performance of individual centres and core labs
- Safety of participants, and notification of and referral for abnormal findings

Further details on the reporting structure of the DSMB can be found within the DSMB Charter in the Supplementary file.

### Adverse event reporting and harms {22}

Information on all adverse events (AEs) will be recorded on the adverse event form. All clearly related signs, symptoms and results of diagnostic procedures performed because of an AE will be grouped together and recorded as a single diagnosis. If the AE is a laboratory abnormality that is part of a clinical condition or syndrome, it will be recorded as the syndrome or diagnosis rather than the individual laboratory abnormality. Each AE will also be described in terms of duration (start and stop date), severity, association with the study product, action(s) taken and outcome.

#### Severe adverse events (SAEs)

Any AE that meets a protocol-defined criterion as a SAE will be submitted immediately (within 24 h of site awareness) on serious adverse event form to the designated Pharmacovigilance Group at the Lagos State Ministry of Health, the LASUTH HREC and NAFDAC. Other supporting documentation of the event may be requested by the designated Pharmacovigilance Group and will be provided as soon as possible. The designated Medical Monitor will review and assess the SAE for regulatory reporting and potential impact on study subject safety and protocol conduct.

At any time after completion of the study, if the PI or appropriate co-investigator becomes aware of an SAE, the PI or appropriate co-investigator will report the event to the designated Pharmacovigilance Group.

#### Reporting events to participants

Subjects will be informed of any severe AEs or SAEs that occur as part of their participation in this trial.

#### Unanticipated problem (UP) reporting

To satisfy the requirement for prompt reporting, unanticipated problems (UPs) will be reported using the following timeline:

- UPs that are SAEs will be reported to the HREC and NAFDAC within 24 h of the investigator becoming aware of the event per the above describe SAE reporting process.
- Any other UP will be reported to the HREC within 3 days of the investigator becoming aware of the problem.

#### Reporting unanticipated problems to participants

Subjects will be informed of any UPs that occur as part of their participation in this trial.

Collection forms for AEs and SAEs can be seen in the CRF forms under the Supplementary files.

### Frequency and plans for auditing trial conduct {23}

The Trial Committee and Data Monitoring Board will meet every 6 weeks. The meeting of the TC and DSMB will be independent of the investigators and the study sponsor.

### Plans for communicating important protocol amendments to relevant parties (e.g. trial participants, ethical committees) {25}

Funders, sponsors and LASUTH HREC will be notified routinely and appropriate approvals gained and communicated as required by the research protocol and by the trial sponsor.

## Dissemination plans {31a}

Findings from the study will be disseminated to local and international conference presentations, as well as scientific peer-reviewed journals for publication. A report will also be submitted to the COVID-19 Presidential Task Force and the Lagos State Government through the Lagos State Ministry of Health with key findings from the study and policy implications.

## Discussion

This study is a double-blind randomized controlled trial designed to provide rigorous evidence on the efficacy of chloroquine phosphate, hydroxychloroquine sulphate and lopinavir/ritonavir compared to standard of care only in the treatment of COVID-19. Despite the promising results from in vitro studies, chloroquine has not been shown conclusively to be efficacious in the treatment of COVID-19. However, it is now part of the recommendations for treatment in several countries while awaiting results of planned and on-going clinical trials [23, 36]. These recommendations have been informed by two main sources—an expert consensus on chloroquine phosphate for treatment of COVID-19 from China and a clinical study undertaken in France [26, 37]. These and one other small study reported from China suggest that chloroquine and hydroxychloroquine prevent worsening of pneumonia, facilitate clearance of the virus and shorten the duration of COVID-19 [24–26, 37]. Broadly, this study is asking three different questions, namely (i) does chloroquine phosphate safely improve patient outcome when added to the standard treatment for COVID-19 compared to the addition of placebo; (ii) does hydroxychloroquine sulphate safely improve patient outcome when added to the standard treatment for COVID-19 compared to the addition of placebo?; and (iii) does lopinavir/ritonavir safely improve patient outcome when added to the standard treatment for COVID-19 compared to the addition of placebo.

The study is being undertaken in an emergency context due to the on-going COVID-19 pandemic. In Nigeria, the number of cases identified is rising as testing capacity has increased and deaths are now being recorded [7]. Lagos State accounts for the largest proportion of cases. The current study is of strategic importance for the State in potentially curbing the burden of COVID-19 disease.

Should the current study demonstrate that either of the three intervention drugs is more efficacious than standard therapy alone, the State Ministry of Health will incorporate this in a standard evidence-based guideline for local management of COVID-19. It is anticipated that such a guideline is likely to be adopted nationally.

## Trial status

The submitted protocol is LACTT version 2, May 15, 2020. Recruitment began on June 15, 2020. The approximate date of recruitment completion is November 21, 2021. The trial protocol can be accessed on the Pan African Clinical Trial Register with number PACTR202004801273802.

## Supplementary Information

**Additional file 1.** Research Consent form.

**Additional file 2.** Case Reporting Forms.

**Additional file 3.** DSMB Charter.

## Abbreviations

AE: Adverse event
ALT: Alanine aminotransferase
CBC: Complete blood count
CNS: Central nervous system
COVID-19: Coronavirus disease 2019
CRF: Case report forms
CTU: COVID-19 treatment unit
DSMB: Drug Safety Monitoring Board
GCP: Good Clinical Practice
GP: Glycoprotein
HCW: Health care worker
HREC: Health Research and Ethics Committee
ICH: International Council for Harmonisation
IgG: Immunoglobulin G
IgM: Immunoglobulin M
IV: Intravenous
IRB: Institutional Review Board
kg: Kilogramme
Mab: Monoclonal antibody
MEURI: Monitored Emergency Use of Unregistered and Investigational Interventions
MoH: Ministry of Health
mg: Milligramme
ml: Millilitre
NIMR: National Institute for Medical Research
PK: Pharmacokinetic
PMT: Project Management Team
RCT: Randomized controlled trial
RNA: Ribonucleic acid
RT-PCR: Reverse transcription-polymerase chain reaction
SAE: Serious adverse event
SAP: Statistical Analysis Plan
SARS: Severe acute respiratory syndrome
SARS-CoV-2: Severe acute respiratory syndrome coronavirus disease 2019
SDCC: Statistical and Data Coordinating Centre
SDSP: Study Data Standardization Plan
SNP: Single nucleotide polymorphisms
SOA: Schedule of activities
SOC: Standard of care
SUSAR: Serious and unexpected suspected adverse reaction
TC: Trial Committee
UP: Unanticipated problem
UPnonAE: Unanticipated problem that is not an adverse event
WBC: White blood cell
WHO: World Health Organization
