# Supplementary material for: A multi-centre, randomized, double-blind, placebo-controlled clinical trial of the efficacy and safety of chloroquine phosphate, hydroxychloroquine sulphate and lopinavir/ritonavir for the treatment of COVID-19 in Lagos State: study protocol for a randomized controlled trial
Source: Trials. 2021 Dec 4;22:869. doi: 10.1186/s13063-021-05675-x (PMC8642768; doi:10.1186/s13063-021-05675-x)
Supplement: Supplementary file 1 — Additional file 1. Research Consent form. [file 13063_2021_5675_MOESM1_ESM.docx]

# **Research Consent form**

**Research Study Approval:**

- The Lagos State University Teaching Hospital Health Research Ethics Committee
- The National Agency for Food and Drug Administration and Control (NAFDAC)

**Identification number for this study**: LREC/06/10/1344

**Study Title:** A Multi-Center, Randomized, Double-Blind, Placebo-Controlled Clinical Trial of the Efficacy and Safety of Chloroquine Phosphate, Hydroxychloroquine sulphate and Lopinavir/Ritonavir for the Treatment of COVID-19 in Lagos State.

**Statement of person obtaining informed consent:** I have fully explained this research to ................................................................. and have given sufficient information, including risks and benefits, to make an informed decision.

**Date**: ................. **Signature:** ...........................  **Name:** ……….......................................

**Statement of person giving consent:** I have been invited to participate in the research on the “*A Multi-Center, Randomized, Double-Blind, Placebo-Controlled Clinical Trial of the Efficacy and Safety of Chloroquine Phosphate, Hydroxychloroquine sulphate and Lopinavir/Ritonavir for the Treatment of COVID-19 in Lagos State*” I have been informed that the risks are minimal, and I am aware that there may be no benefit to me personally. I have been provided the name of the researcher who can easily be reached.

I have read the description of the research or it has been read to me or have had it translated into the language I understand. I have had the opportunity to ask questions about it and the questions have been answered to my satisfaction. I know enough about the purpose, method, risks and benefits of the research. I consent voluntarily to participate in this research and understand that I have the right to withdraw from the research at any time without it in any way affecting my medical care. I have received a copy of this consent form and additional information sheet to keep for myself.

**Date**: ...................... **Signature:** .............................  **Name**: …...........................................

**Witness Signature:** .............................. **Witness Name:** ....................................................
