# Supplementary material for: A multi-centre, randomized, double-blind, placebo-controlled clinical trial of the efficacy and safety of chloroquine phosphate, hydroxychloroquine sulphate and lopinavir/ritonavir for the treatment of COVID-19 in Lagos State: study protocol for a randomized controlled trial
Source: Trials. 2021 Dec 4;22:869. doi: 10.1186/s13063-021-05675-x (PMC8642768; doi:10.1186/s13063-021-05675-x)
Supplement: Supplementary file 2 — Additional file 2. Case Reporting Forms. [file 13063_2021_5675_MOESM2_ESM.docx]

**Case Report Forms**

**Lagos COVID-19 Chloroquine Treatment Trial**

**May 2020**

# Inclusion/Exclusion Criteria

**Lagos COVID-19 Treatment Trial**

**Site:**

**Participant_ID:**

**Visit Date:**

/ / .

d d m m y y y y

**Visit Type:**
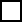
 **Screening**
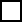
 **Baseline**

## Inclusion Criteria

Participant must: All inclusion criteria must be answered YES to be included in study

1. Agrees to the collection of OP swabs, sputum, and venous blood
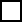
 Yes
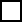
 No
2. Male or non-pregnant female adult ≥18 years of age at time of enrolment
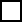
 Yes
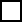
 No
3. Has laboratory-confirmed SARS-CoV-2 infection as determined by PCR, or other assay in any specimen < 72 hours prior to randomisation
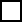
 Yes
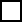
 No
4. Women of childbearing potential must agree to use at least one primary form of contraception for the duration of the study
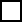
 Yes
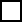
 No
5. Provides written informed consent, understands and agrees to comply with planned study procedures
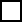
 Yes
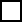
 No

## Exclusion Criteria

Participant must not: All exclusion criteria must be answered NO to be included in study

1. ALT/AST > 5 times the upper limit of normal
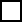
 Yes
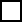
 No
2. Anticipated transfer to another facility which is not a study site within 72 hours
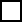
 Yes
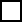
 No
3. Patients with hematological diseases/ chronic liver and kidney disease and reaching end-stage (eGFR < 30) / with arrhythmia and chronic heart disease/ retinal disease, hearing loss or hearing loss/ mental illness/ Skin disorders
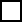
 Yes
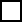
 No
4. Allergy to any chloroquine, hydroxychloroquine or Lopinavir/ ritonavir.
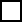
 Yes
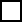
 No
5. Did the participant meet the eligibility requirements for this study?
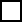
 Yes
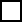
 No

Name and Signature of research staff: …………………………………………..

# **Randomization and Enrollment Form**

**Lagos COVID-19 Treatment Trial L**

**Protocol Number:**

**Site Name:**

**Participant_ID:**

**Form Completion Date:**

/ / .

d d m m y y y y

Is the participant eligible for the study based on inclusion and exclusion criteria?


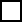
 Yes
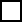
 No **(If no, leave the rest of the form blank.)**

If yes:

1. Date enrolled (signed informed consent form):

/ / .

d d m m y y y y

1. Date all eligibility criteria met:

/ / .

d d m m y y y y

1. Date randomized:

/ / .

d d m m m y y y y

or

1. If eligible and not randomized, indicate reason:* [Optional]


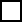
 Failed to return
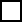
 Declined participation
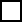
 Other (specify):

Name and Signature of research staff: …………………………………………..

# **Demographics**

**Lagos COVID-19 Treatment Trial**

**Site:**

**Participant_ID:**

**Date:**

/ / .

d d m m m y y y y

**Visit Type:**
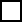
 **Screening**
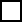
 **Baseline**

1. Gender:
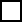
 Female
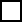
 Male
2. Date of Birth: / / .

d d m m y y y y

1. Nationality (“X” ONLY one with which you MOST CLOSELY identify):


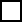
 Nigerian

- Other: ………………………… (specify)

1. Local government and State of primary residence in Nigeria (“X” ONLY one with which you MOST CLOSELY identify):

…………………………………………. [L.G.A]/ ……………………………….. [State]

1. History of International travel in on or after October 2019: a. Yes [] b. No []
2. If “Yes”, Indicate country of travel.

……………………………………….

1. If “Yes”, Indicate date of return into Nigeria: / / 20__ _
2. Any History of physical contact with any individual with a history of International travel in on or after October 2019: a. Yes [] b. No [] c. I don’t know
3. Any History of physical contact with a suspected or lab confirmed COVID-19 case: a. Yes [] b. No [] c. I don’t know
4. If “Yes”: Kindly specify a. suspected COVID-19 case [] b. Lab confirmed COVID-19 case []

Date Informed Consent Signed: / / .

d d m m y y y y

Investigator Signature: / / ..

d d m m y y y y

Medical History

**Lagos COVID-19 Treatment Trial**

**Site Number:**

**Participant_ID:**

**Date:**

/ / .

d d m m y y y y

**Visit Type:**
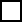
 **Screening**
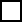
 **Baseline**

Record all past and/or concomitant medical conditions or surgeries. Record only one condition or surgery per line, using the codes provided in the table below. When recording a condition and surgery related to that condition use one line for the condition and one line for the surgery.

01 Head, Eye, Ear, Nose, Throat, Oral

02 Respiratory

03 Cardiovascular

04 Gastrointestinal

05 Genitourinary

06 Musculoskeletal

07 Neurological

08 Endocrine/Metabolic

09 Blood/Lymphatic

10 Dermatologic

11 Psychiatric

12 Allergy

91 Other

| **Code** | **Condition/Disease (one item per line)** | **Start Date  dd/mmm/yyyy** |  | **Current / Resolved** |
| --- | --- | --- | --- | --- |
|  |  |  |  | 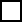 Current 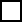 Resolved |
|  |  |  |  | 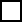 Current 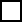 Resolved |
|  |  |  |  | 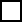 Current 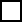 Resolved |
|  |  |  |  | 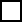 Current 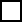 Resolved |
|  |  |  |  | 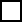 Current 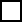 Resolved |
|  |  |  |  | 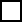 Current 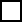 Resolved |
|  |  |  |  | 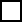 Current 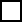 Resolved |
|  |  |  |  | 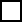 Current 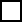 Resolved |
|  |  |  |  | 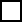 Current 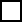 Resolved |
|  |  |  |  | 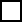 Current 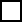 Resolved |

**Instructions**: Add extra rows if needed

Physician: …………………………………………………….. Name& Signature

Physical Exam

**Lagos COVID-19 Treatment Trial**

**Site:**

**Participant_ID:**

**Date:**

/ / .

d d m m y y y y

**Visit Type:**
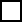
 **Baseline**
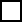
 **Day 8**
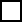
 **Day 29 [Completion visit]**

**Day 3**
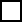
 **Day 11**


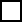


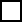
 **Day 5**
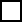
 **Day 15**

| **Category** | **Normal or Abnormal** | **If abnormal, describe below** | **Change from baseline** |
| --- | --- | --- | --- |
| **General Appearance** | 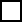 Normal  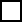 Abnormal  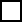 Not Examined |  | 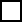 Yes  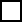 No  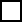 NA |
| **H-E-E-N-T (Head, Eye, Ear, Nose, Throat, Oral)** | 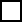 Normal  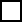 Abnormal  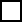 Not Examined |  | 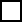 Yes  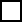 No  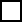 NA |
| **Oral Cavity** | 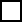 Normal  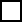 Abnormal  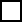 Not Examined |  | 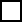 Yes  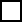 No  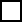 NA |
| **Neck** | 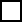 Normal  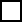 Abnormal  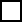 Not Examined |  | 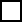 Yes  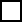 No  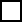 NA |
| **Chest and Lungs** | 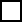 Normal  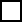 Abnormal  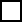 Not Examined |  | 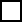 Yes  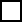 No  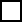 NA |
| **Cardiovascular** | 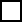 Normal  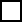 Abnormal  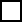 Not Examined |  | 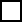 Yes  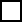 No  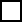 NA |
| **Abdomen** | 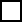 Normal  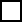 Abnormal  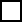 Not Examined |  | Yes  No  NA |
| **Genitourinary** | Normal  Abnormal  Not Examined |  | Yes  No  NA |
| **Rectal** | Normal  Abnormal  Not Examined |  | Yes  No  NA |
| **Musculoskeletal** | Normal  Abnormal  Not Examined |  | Yes  No  NA |
| **Lymph Nodes** | Normal  Abnormal  Not Examined |  | Yes  No  NA |
| **Extremities/ Skin** | Normal  Abnormal  Not Examined |  | Yes  No  NA |
| **Neurological** | Normal  Abnormal  Not Examined |  | Yes  No  NA |
| **Other, specify:  ___________** | Normal  Abnormal  Not Examined |  | Yes  No  NA |

**Note:** *For follow-up PE, if a body system category changes from “Normal” at baseline to “Abnormal” at follow-up due to a new disease/condition or if a preexisting disease/condition worsens from the baseline, an adverse event form should be completed to report the change.*

Physician Name & Signature:

Date signed: / / .

d d m m m y y y y

Vital Signs

**Lagos COVID-19 Treatment Trial**

**Site:**

**Participant_ID:**

**Date:**

/ / .

d d m m y y y y

**Visit Type:**  **Baseline**  **Day 8**  **Day 29**

**Day 3**  **Day 11**  **Completion Visit**

**Day 5**  **Day 15**

1. Time: **:** am pm
2. Heart Rate:  bpm Not done
3. Blood Pressure:  / mmHg (systolic/diastolic) Not done
4. BP Position: Sitting

Supine

Standing

1. Temperature:   °C Not done
2. Respiratory Rate:  /Min Not done
3. SPO2: /% Not done
4. Weight:  Pounds Kilograms Estimated? Not done
5. Height:  Inches Centimeters Estimated? Not done

Physician: ……………………………………………….. [Name & Signature]

Date signed: _____/ _______/ ________ .

d d m m y y y y

Baseline Checklist

**Lagos COVID-19 Treatment Trial**

**Site Name:**

**Participant_ID:**

**Date:**

/ / .

d d m m y y y y

1. Did the participant attend this visit? Yes (if yes, continue) No
2. Please check all assessments completed at this visit:

Demographics

Medical History

Vital Signs

- Physical Exam
- Laboratory Investigations
- Radiologic Investigations (ECG and Chest Xray)

Current Diagnoses and Symptoms

Concomitant Medications (Note: Prior/ Concomitant Medication if protocol proscribes certain medications for a specific period before enrollment)

Inclusion/Exclusion Criteria

Randomization and Enrollment

1. Is the participant continuing in the study? Yes No

If no, remember to complete a **STUDY COMPLETION** form.

If yes, schedule next visit.

**Comments**:

Name & Signature: Date:

Laboratory and Radiological Exam

**Lagos COVID-19 Treatment Trial**

**Site:**

**Participant_ID:**

**Date:**  / / .

d d m m y y y y

**Visit Type:**  **Baseline**  **Day 8**  **Day 29 [Completion Visit]**

**Day 3**  **Day 11**

**Day 5**  **Day 15**

| **Category** | **Normal or Abnormal** | **Values and notes from results, state below** | **Change from baseline** |
| --- | --- | --- | --- |
| **Chest Xray** | [ ] Normal \| [ ] Abnormal \| **[X]** |  | [ ] Yes \| [ ] No \| **[X]** NA |
| **ECG** | [ ] Normal \| [ ] Abnormal \| **[X]** |  | [ ] Yes \| [ ] No \| **[X]** NA |
| **SARS-COV-2 result** | [ ] Normal \| [ ] Abnormal \| **[X]** |  | [ ] Yes \| [ ] No \| **[X]** NA |
| **Serum Creatinine** | [ ] Normal \| [ ] Abnormal \| **[X]** |  | [ ] Yes \| [ ] No \| **[X]** NA |
| **Serum Sodium** | [ ] Normal \| [ ] Abnormal \| **[X**] |  | [ ] Yes \| [ ] No \| **[X]** NA |
| **Serum Potassium** | [ ] Normal \| [ ] Abnormal \| **[X]** |  | [ ] Yes \| [ ] No \| **[X]** NA |
| **CBC with differential** | [ ] Normal \| [ ] Abnormal \| **[X**] |  | [ ] Yes \| [ ] No \| **[X]** NA |
| **Lymphocytes count** | [ ] Normal \| [ ] Abnormal \| **[X]** |  | [ ] Yes \| [ ] No \| **[X]** NA |
| **Lactate dehydrogenase** | [ ] Normal \| [ ] Abnormal \| **[X]** |  | [ ] Yes \| [ ] No \| **[X]** NA |
| **PT / aPTT / INR** | [ ] Normal \| [ ] Abnormal \| **[X]** |  | [ ] Yes \| [ ] No \| **[X]** NA |
| **D- Dimer** | [ ] Normal \| [ ] Abnormal \| **[X]** |  | [ ] Yes \| [ ] No \| **[X]** NA |
| **Albumin** | [ ] Normal \| [ ] Abnormal \| **[X]** |  | [ ] Yes \| [ ] No \| **[X]** NA |
| **Calcium** | [ ] Normal \| [ ] Abnormal \| **[X]** |  | [ ] Yes \| [ ] No \| **[X]** NA |
| **Bicarbonate** | [ ] Normal \| [ ] Abnormal \| **[X]** |  | [ ] Yes \| [ ] No \| **[X]** NA |
| **Chloride** | [ ] Normal \| [ ] Abnormal \| **[X]** |  | [ ] Yes \| [ ] No \| **[X]** NA |
| **Total Bilirubin** | [ ] Normal \| [ ] Abnormal \| **[X]** |  | [ ] Yes \| [ ] No \| **[X]** NA |
| **Total Protein** | [ ] Normal \| [ ] Abnormal \| **[X]** |  | [ ] Yes \| [ ] No \| **[X]** NA |
| **Alanine Aminotransferase [ALT]** | [ ] Normal \| [ ] Abnormal \| **[X]** |  | [ ] Yes \| [ ] No \| **[X]** NA |
| **Alkaline Phosphatase [ALP]** | [ ] Normal \| [ ] Abnormal \| **[X]** |  | [ ] Yes \| [ ] No \| **[X]** NA |
| **Other, specify:  ___________** | [ ] Normal \| [ ] Abnormal \| **[X]** |  | [ ] Yes \| [ ] No \| **[X]** NA |

**Note:** *For follow-up PE, if a body system category changes from “Normal” at baseline to “Abnormal” at follow-up due to a new disease/condition or if a preexisting disease/condition worsens from the baseline, an adverse event form should be completed to report the change.* **[X]** Not examined during visit or not available (NA).

Physician Name & Signature: …………………………………. Date signed: / / .

Medication Monitoring Form

| **Lagos COVID-19 Treatment Trial** | |
| --- | --- |
| **Site Name:___________________________**  **Participant_ID:_________________________** | **This form is cumulative and will be used to capture medications of a single participant throughout the study.** |

**At end of study only: Check this box if participant took no concomitant medication None**

| **Medication [**tick the drug administered**]** | **Time**  **Morning** | **Time**  **Evening** | **Total daily dosage** | **Date** |
| --- | --- | --- | --- | --- |
| IMP1 **[ ]** \| IMP 2 **[ ]** \| \| IMP 3**[ ]** |  |  |  | __/__/ ____ |
| IMP1 **[ ]** \| IMP 2 **[ ]** \| \| IMP 3**[ ]** |  |  |  | __/__/ ____ |
| IMP1 **[ ]** \| IMP 2 **[ ]** \| \| IMP 3**[ ]** |  |  |  | __/__/ ____ |
| IMP1 **[ ]** \| IMP 2 **[ ]** \| \| IMP 3**[ ]** |  |  |  | __/__/ ____ |
| IMP1 **[ ]** \| IMP 2 **[ ]** \| \| IMP 3**[ ]** |  |  |  | __/__/ ____ |
| IMP1 **[ ]** \| IMP 2 **[ ]** \| \| IMP 3**[ ]** |  |  |  | __/__/ ____ |
| IMP1 **[ ]** \| IMP 2 **[ ]** \| \| IMP 3**[ ]** |  |  |  | __/__/ ____ |
| IMP1 **[ ]** \| IMP 2 **[ ]** \| \| IMP 3**[ ]** |  |  |  | __/__/ ____ |
| IMP1 **[ ]** \| IMP 2 **[ ]** \| \| IMP 3**[ ]** |  |  |  | __/__/ ____ |
| IMP1 **[ ]** \| IMP 2 **[ ]** \| \| IMP 3**[ ]** |  |  |  | __/__/ ____ |
| IMP1 **[ ]** \| IMP 2 **[ ]** \| \| IMP 3**[ ]** |  |  |  | __/__/ ____ |
| IMP1 **[ ]** \| IMP 2 **[ ]** \| \| IMP 3**[ ]** |  |  |  | __/__/ ____ |
| IMP1 **[ ]** \| IMP 2 **[ ]** \| \| IMP 3**[ ]** |  |  |  | __/__/ ____ |
| IMP1 **[ ]** \| IMP 2 **[ ]** \| \| IMP 3**[ ]** |  |  |  | __/__/ ____ |

Physician: ……………………………………… Name & Signature Date: __/ __/ ____**Note**: See SOP in appendix for description of IMP 1 (CQ/Pl), 2 (HCQ/Pl) and 3 (LR/Pl)

Concomitant Medications Form

| **Lagos COVID-19 Treatment Trial** | |
| --- | --- |
| **Site Name:___________________________**  **Participant_ID:_________________________** | **This form is cumulative and may be used to capture concomitant medications of a single participant throughout the study.** |

**At end of study only: Check this box if participant took no concomitant medication None**

| **Medication** | **Indication** | **Dosage** | **Start Date** | **Stop Date** | **Ongoing?** |
| --- | --- | --- | --- | --- | --- |
|  |  |  |  |  |  |
|  |  |  |  |  |  |
|  |  |  |  |  |  |
|  |  |  |  |  |  |
|  |  |  |  |  |  |
|  |  |  |  |  |  |
|  |  |  |  |  |  |
|  |  |  |  |  |  |
|  |  |  |  |  |  |
|  |  |  |  |  |  |
|  |  |  |  |  |  |
|  |  |  |  |  |  |
|  |  |  |  |  |  |
|  |  |  |  |  |  |
|  |  |  |  |  |  |
|  |  |  |  |  |  |

Physician: ………………………………………….. Name & Signature

Date: __/ __/ ____

Prior Medications Form

| **Lagos COVID-19 Treatment Trial** | |
| --- | --- |
| **Site Name:___________________________**  **Participant_ID:_________________________** | **This form is cumulative and may be used to capture prior medications of a single participant taken 30 days prior to enrollment.** |

**At end of study only: Check this box if participant took no concomitant medication None**

| **Medication** | **Indication** | **Dosage** | **Start Date** | **Stop Date** | **Ongoing?** |
| --- | --- | --- | --- | --- | --- |
|  |  |  |  |  |  |
|  |  |  |  |  |  |
|  |  |  |  |  |  |
|  |  |  |  |  |  |
|  |  |  |  |  |  |
|  |  |  |  |  |  |
|  |  |  |  |  |  |
|  |  |  |  |  |  |
|  |  |  |  |  |  |
|  |  |  |  |  |  |
|  |  |  |  |  |  |
|  |  |  |  |  |  |
|  |  |  |  |  |  |
|  |  |  |  |  |  |
|  |  |  |  |  |  |
|  |  |  |  |  |  |

Physician: ………………………………………….. Name & Signature Date: __/ __/ ____

| **Lagos COVID-19 Treatment Trial** | |
| --- | --- |
| **Site Name:___________________________**  **Participant_ID:_________________________** | **This form is cumulative and captures adverse events of a single participant throughout the study.** |

| **Severity** | **Study Intervention Relationship/ Causality assessment** | **Action Taken Regarding Study Intervention** | **Outcome of AE** | **Expected** | **Serious Adverse Event (SAE)** |
| --- | --- | --- | --- | --- | --- |
| 1 = Mild  2 = Moderate  3 = Severe  4 = Life-Threatening | 0 = Not related  1 = Unlikely related  2 = Possibly related  3 = Probably related  4 = Definitely related | 0 = None  1 = Dose modification  2 = Medical Intervention  3 = Hospitalization  4 = Intervention discontinued  5 = Other | 1 = Resolved  2 = Recovered with minor sequelae  3 = Recovered with major sequelae  4 = Ongoing/Continuing treatment  5 = Condition worsening  6 = Death  7 = Unknown | 1 = Yes  2 = No | 1 = Yes  2 = No  (if yes, complete SAE form) |

**At end of study only: Check this box if participant had no adverse events None**

| **Adverse Event** | **Start Date/ time** | **Stop Date/ time** | **Severity** | **Relationship** | **Action Taken** | **Outcome of AE** | **Expected?** | **SAE?** |
| --- | --- | --- | --- | --- | --- | --- | --- | --- |
|  |  |  |  |  |  |  |  |  |
|  |  |  |  |  |  |  |  |  |
|  |  |  |  |  |  |  |  |  |
|  |  |  |  |  |  |  |  |  |
|  |  |  |  |  |  |  |  |  |
|  |  |  |  |  |  |  |  |  |
|  |  |  |  |  |  |  |  |  |
|  |  |  |  |  |  |  |  |  |
|  |  |  |  |  |  |  |  |  |

Name and Signature of Physician: …………………………………………..

**Study Completion**

**Lagos COVID-19 Treatment Trial**

**Site Number:**

**Participant_ID:**

**Visit Date:**

/ / .

d d m m y y y y

1. Date of final study visit: / / .

d d m m y y y y

1. Date of last-known study intervention: / / .

d d m m y y y y

1. Primary reason for terminating participation in the study:

Completed study

Participant was determined after enrollment to be ineligible (provide comments):

Participant withdrew consent

In the principal investigator’s opinion, it was not in the participant’s best interest to continue (provide comments):

Adverse event (If checked, complete the AE form.)

Death

Lost to follow-up

Other (specify):

Unknown

**Comments**:

Principal Investigator Signature: Date:

Study Exit Checklist

**Lagos COVID-19 Treatment Trial**

**Site Name:**

**Participant_ID:**

**Date:**

/ / .

d d m m y y y y

1. Did the participant attend this visit? Yes (if yes, continue) No
2. Please check all assessments completed at this visit:

Demographics

Medical History

Vital Signs [days 1-15]

- Physical Exam
- Laboratory Investigations [day 1, 3, 5, 8. 11 and 15]
- Radiologic Investigations (ECG and Chest Xray)

Current Diagnoses and Symptoms

- Concomitant Medications (Note: Prior/ Concomitant Medication if protocol proscribes certain medications for a specific period before enrollment)

3. Did this participant complete the study? Yes No

If no, remember to indicate the reason why: ………………………………………………………...

…………………………………………………………………………………………………………

**Comments**:

Name & Signature: Date:
