# Supplementary material for: A multi-centre, randomized, double-blind, placebo-controlled clinical trial of the efficacy and safety of chloroquine phosphate, hydroxychloroquine sulphate and lopinavir/ritonavir for the treatment of COVID-19 in Lagos State: study protocol for a randomized controlled trial
Source: Trials. 2021 Dec 4;22:869. doi: 10.1186/s13063-021-05675-x (PMC8642768; doi:10.1186/s13063-021-05675-x)
Supplement: Supplementary file 3 — Additional file 3. DSMB Charter. [file 13063_2021_5675_MOESM3_ESM.docx]

**DATA AND SAFETY MONITORING BOARD CHARTER**

**INTRODUCTION**

This Charter is for the Data and Safety Monitoring Board (DSMB) for the study titled ‘**A Multi-Center, Randomized, Double-Blind, Placebo-Controlled Clinical Trial of the Efficacy and Safety of Chloroquine Phosphate, Hydroxychloroquine sulphate and Lopinavir/Ritonavir for the Treatment of COVID-19 in Lagos State.** The Charter is intended to be a living document. The DSMB may wish to review it at regular intervals to determine whether any changes in procedure are needed.

**RESPONSIBILITIES OF THE DSMB**

The DSMB is responsible for safeguarding the interests of study participants, assessing the safety and efficacy of study procedures, and for monitoring the overall conduct of the study

The DSMB is an independent group and is required to provide recommendations about starting, continuing, and stopping the study. In addition, the DSMB is asked to make recommendations, as appropriate about:

- Efficacy of the study intervention
- Benefit/risk ratio of procedures and participant burden
- Selection, recruitment, and retention of participants
- Adherence to protocol requirements
- Completeness, quality, and analysis of measurements
- Amendments to the study protocol and consent forms
- Performance of individual centers and core labs
- Participant safety, and
- Notification of and referral for abnormal findings

**ORGANIZATION AND INTERACTIONS**

Communication with DSMB members will be primarily through the Trial Manager (TM), it is expected that other study investigators will not communicate with DSMB members about the study directly, except when making presentations or responding to questions at DSMB meetings or during conference calls.

**DSMB MEMBERS**

DSMB members and their expertise are listed in Appendix A. The DSMB will have a Chair and Secretary elected by the board members. The Secretary is responsible for assuring the accuracy and timely transmission of the final recommendations and DSMB minutes.

**SCHEDULING, TIMING, AND ORGANIZATION OF MEETINGS**

DSMB meetings are usually held by remote connections. The purpose of the first meeting is to review and discuss this Charter, provide an overview of study activities, review and make recommendations about the protocol(s) presented to it, and to determine the frequency of interim analyses and whether data will or will not be masked to identity of randomized groups. Enrollment in a study cannot begin until the DSMB’s Charter has been accepted by NAFDAC.

For this Chloroquine Treatment Trial, meetings will be held four times; before commencement of the study, 2 meetings monthly, and at the end of the study.

- Review of interim data analyses will occur: _________________________________________________________.

The agenda for DSMB meetings and calls may be drafted by the secretary in consultation with the chairman. The agenda and meeting materials should be distributed by the secretary a week before each meeting or call.

Before each meeting, when the agenda is sent out, the secretary will ask all DSMB members to state whether they have developed any new conflicts of interest. The Chair and other members will determine if the conflict limits the ability of the DSMB member to participate in the discussion. The DSMB also will review adverse event data, other safety data, enrollment data, and quality and completeness of study data at each meeting to ensure proper trial conduct. At intervals, the DSMB will also review formal interim analyses of the primary end point.

It is expected that all DSMB members will attend every meeting and call. However, it is recognized that this may not always be possible. Quorum for voting is considered to be 3 out of the five members. The Board may wish to decide if particular expertise is needed within the quorum for the meeting to be valid. All standing Monitoring Board members are voting members. The Board may also wish to decide in advance whether *ad hoc* members can vote.

**DISCUSSION OF CONFIDENTIAL MATERIAL**

DSMB meetings and calls will be organized into open, closed, and executive sessions.

- During the **open sessions**, information will be presented to the DSMB by the study investigators with time for discussion.
- During the **closed sessions**, the DSMB will discuss confidential data from the study, including information on efficacy and safety by treatment arm. The DSMB will decide whether to remain masked to the treatment assignments at each meeting.

If the **closed session** occurs on a conference call or video connection, steps will be taken to ensure that only the appropriate participants are on the call, and to invite others to re-join the call only at the conclusion of the closed session, after this, participants will be re-convened so that the DSMB Chair can provide a summary of the DSMB’s recommendations. This provides an opportunity for study investigators to ask questions to clarify the recommendations. The meeting is then adjourned.

**REPORTS OF DSMB DELIBERATIONS**

- Formal minutes: The Secretary is responsible for the accuracy and transmission of the formal DSMB minutes. These minutes are prepared to summarize the key points of the discussion and debate, requests for additional information, response of the investigators to previous recommendations, and the recommendations from the current meeting. If concerns are identified, the report will outline the concerns, the board’s discussion of the concerns, and the basis for any recommendations that the DSMB has made in response to the concerns.
- The DSMB Chair may sign the minutes or indicate approval electronically via email. If there are no concerns or major issues raised, signed minutes will be sent to the TM within five (5) days of each meeting or call. If concerns or major issues are raised during the meeting, signed minutes will be sent to the TM within seven (7) days of the meeting or call. The TM will forward the minutes to appropriate AIs and IRBs as soon as possible. Subsequently, minutes are included in the materials for the subsequent DSMB meeting to be approved by voice vote at that meeting. Once they have been voted and approved by the Board, they are considered final.

**REPORTS TO THE DSMB**

For each meeting, TM will prepare summary reports and tables to facilitate the oversight role of the DSMB. The DSMB should discuss at the first or subsequent meetings what data they wish to review and how it should be presented.

**STATISTICAL MONITORING GUIDELINES**

At the first meeting, review of the protocol will include review of the statistical analysis plan. The DSMB should discuss the adequacy of that plan. The DSMB should discuss the statistical monitoring procedures they propose to follow to guide their recommendations about termination or continuation of the trial. These procedures could include guidelines for early termination for benefit, termination for futility, and termination for safety reasons.

**Appendix A** - DSMB MEMBERS AND THEIR EXPERTISE

| S/N | NAME | POST | EXPERTISE |
| --- | --- | --- | --- |
| 1 | Prof. Bayo Onajole | Chairman | Epidemiologist |
| 2 | Prof. O. O. Odusanya | Member | Community Physician |
| 3 | Prof. Ibrahim Oreagba | Member | Clinical Pharmacologist |
| 4 | Dr. Babatunde Saka | Secretary | Infectious Disease Epidemiologist |
| 5 | Dr. Afusat Adesina | Member | Clinical Pharmacist |
